# Effects of Bone Marrow Mesenchymal Stem Cells-Conditioned Medium on Tibial Partial Osteotomy Model of Fracture Healing in Hypothyroidism Rats

**DOI:** 10.22034/ibj.22.2.90

**Published:** 2018-03

**Authors:** Niloofar Sefati, Mohsen Norouzian, Hojjat-Allah Abbaszadeh, Mohammad-Amin Abdollahifar, Abdollah Amini, Mohammad Bagheri, Arefeh Aryan, Fatemeh Fadaei Fathabady

**Affiliations:** 1 Department of Biology and Anatomical Sciences, School of Medicine, Shahid Beheshti University of Medical Sciences, Tehran, Iran; 2 Laser Application in Medical Sciences Research Center, Shahid Beheshti University of Medical Sciences, Tehran, Iran; 3 Hearing Disorders Research Center and Department of Biology and Anatomical Sciences, School of Medicine, Shahid Beheshti University of Medical Sciences, Tehran, Iran; 4 Medical School, Shahid Beheshti University of Medical Sciences, Tehran, Iran

**Keywords:** Conditioned medium, Hypothyroidism, Mesenchymal stem cells, Osteotomy

## Abstract

**Background:**

Hypothyroidism is associated with dysfunction of the bone turnover with reduced osteoblastic bone formation and osteoclastic bone resorption. Mesenchymal stem cells (MSCs) secrete various factors and cytokines that may stimulate bone regeneration. The aim of this study was to determine the effects of MSCs-conditioned medium (CM) in hypothyroidism male rats after inducing bone defect.

**Methods:**

In this study, 24 male rats were randomly assigned to three groups: (I) hypothyroidism + bone defect (HYPO), (II) hypothyroidism + bone defect + CM (HYPO + CM), and (III) no hypothyroidism + bone defect (control). Four weeks after surgery, the right tibia was removed, and immediately, biomechanical and histological examinations were performed.

**Results:**

The results showed a significant reduction in bending stiffness (32.64 ± 3.99), maximum force (14.63 ± 1.89), high stress load (7.59 ± 2.31), and energy absorption (12.68 ± 2.12) at the osteotomy site in hypothyroidism rats in comparison to the control and hypothyroidism + condition medium groups (*p* < 0.05). There was also a significant decrease in the trabecular bone volume (3.86 ± 3.88) and the number of osteocytes (5800 ± 859.8) at the osteotomy site in hypothyroidism rats compared to the control and hypothyroidism + condition medium groups (*p* < 0.01 and *p* < 0.02, respectively).

**Conclusion:**

The present study suggests that the use of the CM can improve the fracture regeneration and accelerates bone healing at the osteotomy site in hypothyroidism rats.

## INTRODUCTION

Thyroid is an endocrine gland that is specialized for the production and storage of thyroid hormones (THs). THs are important mediators of metabolism and energy expenditure in response to environmental demands, and play an important role in growth, development, and bone homeostasis of human and animal skeletal system[1]. Besides, THs induce osteoblasts and osteoclast precursors[2]. It has been revealed that hypothyroidism can induce growth arrest and delay bone development in children[3]. Studies have shown that there is a correlation between hypothyroidism and the dysfunction of the bone turnover, which can affect osteoblastic bone formation and osteoclastic bone resorption. Hypothyroidism has also been indicated to be able to increase the duration of the bone remodeling and causes a delay in mineralization[4,5]. It is obvious that hypothyroidism is associated with increased bone fracture risk[6], but clinical studies have reported that it causes a decrease in osteogenesis at the site of bone defects[7].

Mesenchymal stem cells (MSCs) have high differentiation capacity, and they are ideal sources for effective treatment of bone fracture[8]. In addition, stem cell therapy has been reported to have beneficial effects on bone tissue repair[9]. A previous study has shown that MSCs transplantation could stimulate the new bone callus in bone defect models in animals[10]. The MSCs secrete various factors and cytokines, which can add into conditioned medium (CM) under specific physiological conditions[11]. It has also been demonstrated that growth factors and cytokines secreted from the MSCs regulate tissue repair via several signaling pathways such as Smad and extracellular signal-regulated kinase 1/2 pathways[12]. The paracrine effects of growth factors and cytokines secreted from the MSCs may stimulate tissue regeneration or have anti-apoptotic effects[13]. Human MSC-CM can also stimulate the recruitment of bone marrow endothelial cells and stromal cells to promote angiogenesis, callus formation, and fracture healing in a diabetic model[14,15].

The aim of the present study was the evaluation of the morphological changes, including total volume, trabecular volume, bone marrow volume, cortical bone volume, as well as the numbers of osteoblast, osteocytes, and osteoclast in the bone defects using modern stereological techniques. In the light of the above-mentioned reports, this investigation was designed to explore the effects of MSCs-CM on bone defects induced in hypothyroidism male rat models.

## MATERIALS AND METHODS

### Animals

In total, 24 adult male Wistar rats (200-220 g) were obtained from the laboratory animal center of Shahid Beheshti University of Medical Sciences, Tehran, Iran. The Ethics Committee of the University approved the animal experiment (IR.SBMU. SM. REC.1394.31). The male rats were randomly categorized into three groups: Group I, hypothyroidism + bone defect (HYPO), Group II, hypothyroidism + bone defect + CM (HYPO + CM), and Group III, no hypothyroidism + bone defect (control). Each group included eight rats that were housed under standard conditions (22–24 °C room temperature and a 12:12 h light-dark schedule) and had free access to water and food and digested were given 4 mg powdered methimazole (Sigma, USA) dissolved in 100 cc distilled water for four weeks[16]. THs were determined after methimazole treatment. After obtaining blood samples from the corner of animals’ eyes, THs (T3, T4, and TSH) levels were determined using ELISA kits (DiaPlus, USA). After a latency period of five days of the partial osteotomy procedure, 20 μl CM was injected intraperitonely (five injections in total).

### Partial osteotomy

**Fig. 1:**
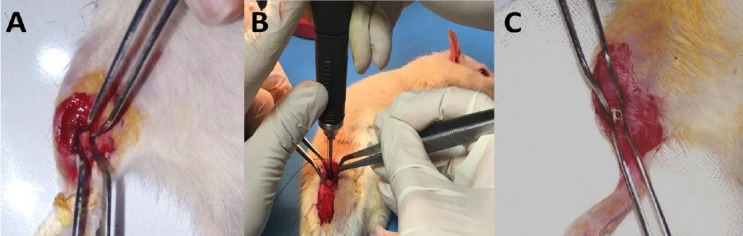
Steps in the partial osteotomy. The Figure shows the incision and exposed tibial midshaft (A), circular partial osteotomy procedure with low-speed drill (B), and partial bone fracture (C)

The skin of the right leg of each rat was cut longitudinally to expose the tibial midshaft. As indicated in Figure 1, a partial osteotomy was created with a low-speed drill (terminal, 1.5 mm diameter; Delab; Dental Fabriktreffurt, Germany)[17].

### Isolation of mesenchymal stem cells and cell culture

MSCs were isolated from 8-week-old male rats. Rats were sacrificed, the femora were dissected out under sterile conditions, and the edge of each bone was cut. DMEM (Gibco, USA) was then injected into the bone marrow using an 18-gauge syringe, and the bone marrow cells were flushed out to the opposite side; this procedure was repeated several times. Afterwards, the bone marrow cells were seeded into a tissue culture flask in DMEM containing an antibiotic-antimitotic solution (100 units/ml penicillin G and 100 mg/ml streptomycin, both from Gibco, USA), and the medium was supplemented with 10% FBS. Three days after seeding, floating cells were removed, and the medium was replaced with a fresh medium. The adherent, spindle-shaped cells were passaged when the cells approached confluence. Adherent cells were collected with Trypsin/EDTA, re-suspended in a fresh medium and transferred to new flasks at a density of 1 × 10^4^ cells/cm^2^. The fixed cells were washed twice with PBS (Sigma, USA) and incubated at 4 °C with antibodies to the following antigens: CD34, CD45, CD90, and CD44 (all from Chemicon, USA) for 30 min. Primary antibodies were directly conjugated with phycoerythrin phycoerythrin. Flow cytometry was performed with a FACS can flow cytometer (Becton Dickinson, USA)[18], and flow cytometry analysis was carried out by a Partec CyFlow Space cytometer using FloMax software.

### Osteogenic and adipogenic differentiation

Cells were seeded in an osteocytogenic differentiation medium containing low-glucose DMEM containing 10% FBS, 0.1 μM dexamethasone (Sigma-Aldrich, USA), 200 μM L-ascorbic acid-2-phosphate (Sigma-Aldrich, USA), and 10 mM β-glycerol phosphate (Sigma-Aldrich, USA) and in an adipogenic medium consisting of DMEM/10% FBS, 50 μmol/l indomethacin, 10 μM insulin, 1 μmol/l dexamethasone, and 0.5 mM 3-isobutyl-1-methyl-xanthine (all from Sigma-Aldrich, USA) for 21 days. Osteocytogenic and adipogenic induction was confirmed by Alizarin Red S and Oil Red staining, respectively[19,20].

### Preparation of mesenchymal stem cells-conditioned media

In order to obtain the MSC-CM, bone marrow mesenchymal stem cells (BMMSCs) at passage four were seeded at a density of 10,000 cells/cm^2^. At 80% confluence, the cells were washed three times with PBS and the media were replaced with serum-free DMEM. After 48 h, cells were incubated and then media were stored at -80 °C until use. For the *in vivo* assays, the CM was concentrated 20fold by lyophilized-drying (Christ Alpha1-2 LD Plus, Germany) according to the manufacturer’s instructions[21].

### Tissue preparation

Histological evaluation was performed at 4 weeks after surgery. Every right tibia was removed, and soft tissues including skins and muscles were eliminated from the tibia. Tissue samples (proximal half of each right tibia including the fractured and defected areas) were fixed in 10% formalin for 48 hours and decalcified in 10% nitric acid. Then the defected areas were embedded in paraffin blocks and cut longitudinally into 5-μm and 25-μm thick sections with a microtome. For the microscopic descriptive analysis of each group, slides were stained by hematoxylin and eosin (H&E) and Masson’s trichrome dyes. Bone healing evaluation was performed using a microscope connected to an image analyzer. All measurements were performed using a magnifying objective (4× and 40×).

### Biomechanical examination

Rats were sacrificed four weeks after the surgery, and the right tibias were then collected and weighed. The biomechanical properties of five tibias from the groups were examined. Bones were subjected to three-point bending on a material testing device (Zwick/Roell Group, Z 2.5 H 15WN, UIm, Germany) until fracture took place in the bone. The entire bones were placed in similar orientation in the testing machine. Two loading points, 19 mm apart, were used to mount each bone; a press head was subsequently activated to squeeze the center of shaft in bones until fracture occurred. The compressive loading speed was 0.08 mm/s during the testing time. Data were automatically recorded by the material testing device, which received the data from the load-deformation curve. The following parameters were computed: bending stiffness (N/mm), energy absorption (N/mm), maximum force (N), and stress high load (N/mm^2^). Bending stiffness is the slope on the linear portion of the load-deformation curve. Energy absorption is the amount of energy absorbed by the bone until breakage. Maximum force is the force needed to break the bone. The stress high load was calculated by dividing N by the surface area (mm^2^) of bone at the osteotomy site[22].

### Stereological study

#### Measurement of bone volume

Using a microscope connected to a camera, volumes of bone trabecular, bone marrow, cortical bone, and fibrous tissue were calculated using the Cavalieri method[23,24] as the product of the areas and measured tissue thickness between the saved sections. Using stereological software, the total area of the sections (∑*A*) of the tibial fracture region was determined, and finally, the volume was estimated by the following formulation[23,24]:


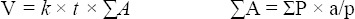


Where “ΣP” is the total points hitting the tissue sections, “a/p” is the area associated with each point, and “k × t” is the distance between the sampled sections.

#### Measurement of total number of the bone cells

For the estimation of numerical density and total number of the bone cells, dissector method was used. Sections were measured with the optical disector. The specimens were evaluated at 40× oil immersion magnification with a high numerical aperture. An image was captured and analyzed by a computer. The focus plane was set at the surface of the specimen. Then a set of three unbiased measurement frames was superimposed on the live image (Fig. 1A). At the same time, the microcator measuring the optical distance through the specimen in the z axis was zeroed. By gently moving the focus down through the specimen, an approximately 0.5-mm thin focal plane made objects come into focus and disappear. Bone cells falling in the measurement frames’ permitted areas were counted as they came into focus until the microcator indicated that the focal plane had traveled 10 µm through the specimen. The numerical density of cells was obtained by the following formulation:


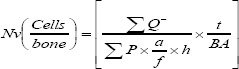


where ΣQ^-^” is the number of the nuclei coming into focus and counted, “ΣP” is the total number of the counting frames in all fields, a/f is the area per frame, “h” is the height of the disector, “t” is the real section thickness measured using the microcator when the Q^-^ was counted, and BA was the block advance of the microtome. To estimate the total number of the bone cells, the following formula was used: N (bone cell) = Nv × V (final)[25,26].

## RESULTS

### Thyroid hormones level

**Fig. 2:**
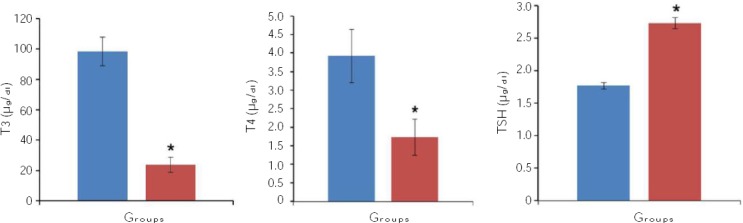
Mean ± SEM of T3 (A), T4 (B), and TSH (C) levels before and after methimazole treatment. There was a significant difference in T3, T4, and TSH levels between methimazole-treated group and control group (*p < 0.05).

Figure 2 shows the result of T3, T4, and TSH levels before and after methimazole treatment. As indicated in the Figure, a significant difference was observed in T3, T4, and TSH levels between methimazole-treated and the control groups.

### Rat BMSCs characterization

**Fig. 3:**
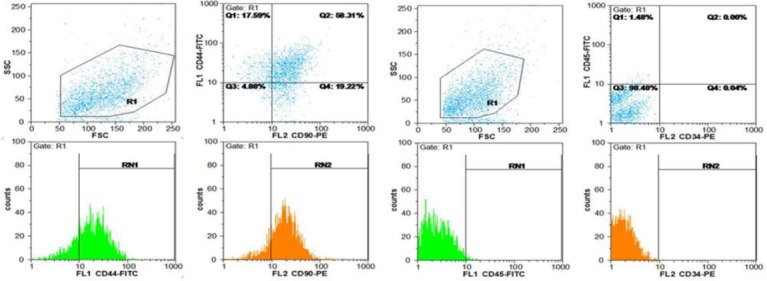
Flow cytometry analysis of passage 3 mesenchymal stem cells culture for CD45, CD34, CD44, and CD90 cells.

The BMSCs appeared as a monolayer of large, fibroblast-like flattened adherent cells at passage 4. Flow cytometry analysis of rat BMSCs within 3-5 passages showed that rat BMSCs were CD90 and CD44 positive, but CD34 and CD45 negative (Fig. 3).

### Confirmation of osteogenic and adipogenic potential

**Fig. 4:**
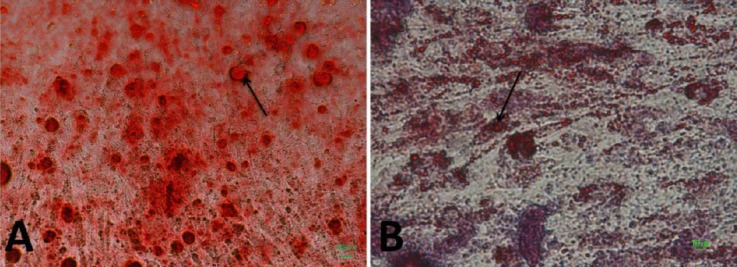
Bone marrow mesenchymal stem cells, osteogenic and adipogenic differentiation. Alizarin Red S staining (A) for mineral deposition was performed. Arrow shows mineral deposition. Oil Red staining (B) was evidenced by the formation of lipid droplets after 21 days. Arrow indicates lipid droplets.

Potential differentiation of MSC into adipocytes and osteocytes was done, and these abilities were proved via Alizarin Red S staining that showed the presence of calcium deposits (Fig. 4), while Oil Red O staining indicated the presence of lipid droplets (Fig. 4).

### Biomechanical results

**Fig. 5:**
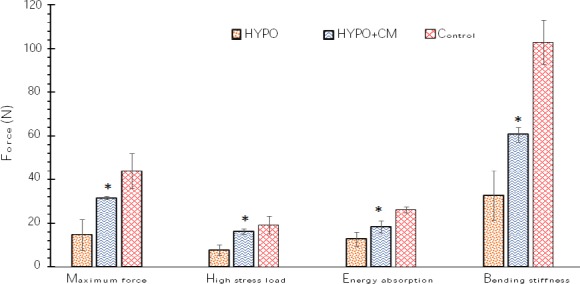
Mean ± SEM of maximum force (N), high stress load (N/mm^2^), energy absorption (N/mm), and bending stiffness (N/mm) in the different groups. *shows the significant difference between HYPO group and the other groups (*p* < 0.05).

As indicated in Figure 5, a significant increase was observed in the bending stiffness (N/mm), maximum force (N), high stress load (N/mm^2^), and energy absorption (N/mm) of bone at the osteotomy site of the control group in comparison to the HYPO and HYPO + CM groups (*p* < 0.05). The data revealed that the use of the CM increases the bending stiffness, maximum force, high stress load, and energy absorption of the HYPO + CM group compared to the HYPO group (Fig. 5).

### Stereological result

**Fig. 6:**
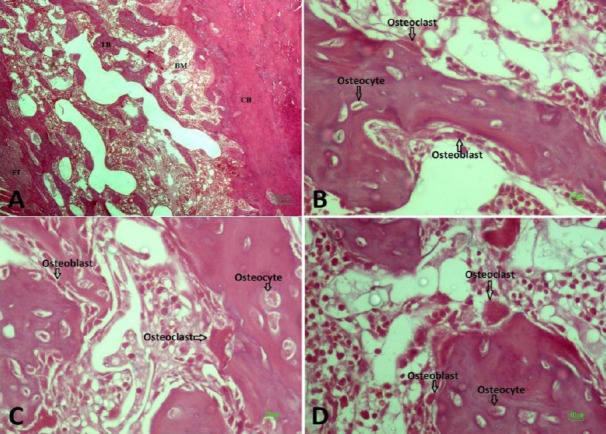
Micrograph of the bone defect stained with H& E. (A) Trabecular bone (TB), bone marrow (BM), fibrous tissue (FT), and cortical bone (CB); (B) Hypothyroidism; (C) Hypothyroidism + CM; (D) Control.

**Fig. 7:**
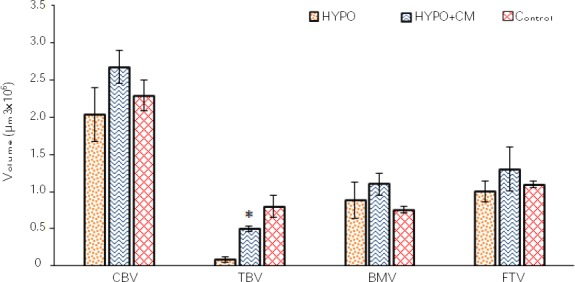
Mean ± SEM of cortical bone volume (CBV), trabecular bone volume (TBV), bone marrow volume (BMV), and fibrous tissue volume (FTV) in different groups. *shows the significant difference between HYPO group and the other groups (*p* < 0.01).

Based on the findings of the current study, the total volumes of the bone, cortical bone, bone marrow, and trabecular bone remained unchanged in the control, HYPO, and HYPO + CM groups (Fig. 3). The data revealed that the use of the CM has no effect on total volumes of bone, cortical bone, bone marrow, and trabecular bone at the osteotomy site in hypothyroidism rats (Figs. 6 and 7).

#### Total numbers of osteoblasts, osteocytes, and osteoclasts

**Fig. 8:**
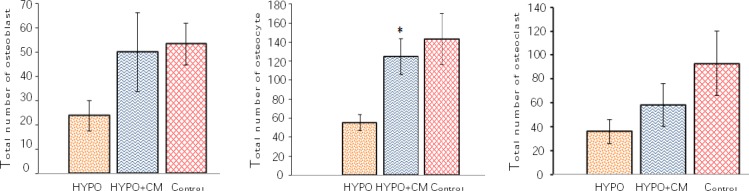
Mean ± SEM of number of osteoblasts (A), osteocytes (B), and osteoclasts (C) in different groups. * indicates the significant difference between HYPO group and the other groups (*p* < 0.02).

According to the findings of the current study, the volume of the bone marrow remained unchanged in the control group, HYPO group and HYPO + CM groups (Figs. 6 and 8). The results showed that a significant decrease was observed in the total number of the osteocyte of bone at the osteotomy site of the HYPO group in comparison to the control and HYPO + CM groups (*p* < 0.02). However the study of the data obtained from energy absorption analysis showed that the use of the CM increases the total number of the osteocyte of the HYPO + CM group compared to the HYPO group (Figs. 6 and 8). The results also showed that the number of the osteoclast remained unchanged in the control, HYPO, and HYPO + CM groups (Figs. 6 and 8).

## DISCUSSION

The present study evaluated the effects of bone marrow MSCs-CM collected from BMMSCs on tibial partial osteotomy model in hypothyroidism male rats using stereological methods. Our results revealed that treatment with methimazole at the first step could induce hypothyroidism in rats and showed a significant reduction in the volume of trabecular bone and the number of osteocytes in defected bone regions. Thyroid diseases have some effects on bone metabolism due to the change in the metabolic actions. Fracture risk is significantly increased in hypo-thyroidism; however, hypothyroidism has a significant effect on bone mineral metabolism. The THs participate in bone mineral homeostasis and bone mineral density[27]. The hypothyroidism is associated with bone remodeling delay[7]. These findings are in agreement with the previous studies showing the adverse effects of hypothyroidism on the bone tissue.

Vestergaard *et al*.[28] reported that fracture risk was mainly increased after the diagnosis of hypothyroidism in the age group up to 50 years. Polovina *et al*.[29] noticed that hypothyroidism increased the risk of low-trauma hip fracture in the postmenopausal patients. It should be noted that osteogenesis activity is decreased in defected bone regions in hypothyroid rats[7,30,31]. Our results showed significant differences in biomechanical and stereological parameters after tibial partial osteotomy in the hypothyroidism group. The results also indicated a significant increase in the bending stiffness, maximum force, high stress load, and energy absorption in the hypothyroidism group. Evaluation of maximum force of the bones was done using a three-point bending test on bone pressure resistance. For evaluation of biomechanical strength in animal models, the bending test is a valid method[16,21]. The stereological results showed that hypothyroidism significantly decreased trabecular bone volume and the number of osteocytes after tibial partial osteotomy. The advantage of using stereological techniques in this study is obtaining unbiased and accurate estimations[23,24,32]. Additionally, treatment with CM has regenerative potential in bone defect models.

Stem cell therapy has beneficial effects on bone tissue regeneration[9]. Some studies have shown the role of stem cells in tissue regeneration and healing the release of paracrine factors such as angiogenic and osteogenenic factors. There is little information about the mechanism of CM in the bone regeneration, but its effect on the bone defect may involve the formation of release of paracrine factors such as vascular endothelial growth factor and monocyte chemoattractant protein 1[13,33,34]. Previous reports have suggested that angiogenesis and osteogenesis can be promoted by CM collected from BMMSCs, and also CM could be a stimulator for blood vessels and bone formation[35-37]. However, formation of blood vessels plays a key role in bone formation because blood vessels can transport oxygen, soluble growth factors, nutrients, and various types of cells. Fujio and co-workers[37] evaluated the angiogenic and regenerative potential of CM from human dental pulp cells on bone healing during distraction osteogenesis. They suggested that the vascular endothelial growth factor-A and Ang-2 released from human dental pulp cells promoted angiogenesis in bone defect, thereby improving and accelerating bone healing[37]. The effects of CM on bone tissue regeneration observed in our study may result from its angiogenesis and osteogenesis properties. The current study also suggests that CM can increase bone formation through trabecular bone formation in bone defect region. This investigation also confirmed that CM from BMMSCs could improve the bone repair procedure, possibly through the action of secreted growth factors and can reduce the healing time in a partial osteotomy model.
